# Bending Response of Cross-Ply Laminated Composite Plates with Diagonally Perturbed Localized Interfacial Degeneration

**DOI:** 10.1155/2013/350890

**Published:** 2013-11-10

**Authors:** Chee Zhou Kam, Ahmad Beng Hong Kueh

**Affiliations:** Construction Research Centre, Universiti Teknologi Malaysia (UTM-CRC), 81310 Johor Bahru, Johor, Malaysia

## Abstract

A laminated composite plate element with an interface description is developed using the finite element approach to investigate the bending performance of two-layer cross-ply laminated composite plates in presence of a diagonally perturbed localized interfacial degeneration between laminae. The stiffness of the laminate is expressed through the assembly of the stiffnesses of lamina sub-elements and interface element, the latter of which is formulated adopting the well-defined virtually zero-thickness concept. To account for the extent of both shear and axial weak bonding, a degeneration ratio is introduced in the interface formulation. The model has the advantage of simulating a localized weak bonding at arbitrary locations, with various degeneration areas and intensities, under the influence of numerous boundary conditions since the interfacial description is expressed discretely. Numerical results show that the bending behavior of laminate is significantly affected by the aforementioned parameters, the greatest effect of which is experienced by those with a localized total interface degeneration, representing the case of local delamination.

## 1. Introduction

In recent years, fiber reinforced laminated composite materials have seen popularity in various extreme engineering and structural applications. Exceptional performance of the materials, which is a direct product of their high stiffness- and strength-to-weight ratios, makes them recognizable as one of the highly demanded advanced materials. One particular attraction that guides to their paramount application owes to tunable properties which can be achieved desirably in accordance with proper design. In spite of such attractiveness, it is broadly acquainted that defects in laminated composites, which occur during their manufacturing process and service life, are unavoidable events. These defects lead to the degradation of stiffness and rigidity and, as a result, a compromised performance prior to the intended design life. Of all potential contributors, the behavior discount coming from the defect in terms of interfacial bond degeneration is particularly of high significance due to the layered nature of laminated composites. Therefore, the influence of degenerated bonding on the mechanical response of laminated composite, which constitutes the subject of this paper, has continually been considered as one of the principal concerns in the analysis and design processes to prohibit an overestimation of the performance of the material in practice.

To appreciate the current advances in our undertaken subject, we present next a brief account of existing works in connection with the interfacial degeneration behaviors. Early works on the imperfect bonding had been focused on the shear slip in cross-ply laminates adopting Pagano's analytical solutions [1–3], due to limited experimental investigation, as highlighted by Murakami [4]. Toledano and Murakami [5] considered the shear slip in a two-layer cross-ply composite laminate (length-to-thickness ratio, *S* = 6) through the inclusion of both linear and nonlinear interface slip laws, which had been proven to be valid in analyzing the beam with an interlayer slip [4]. The bending response of a laminate with the same stacking sequence had been examined by Lu and Liu [6] using the interlayer shear slip theory (ISST) and others [7–10] using the linear spring-layer model, in which the midplane deflection under a variety of shear slip coefficients as well as through-thickness midpoint deflection was addressed in [6–8] as well as Shu and Soldatos [9, 10], respectively. Da Silva and Sousa Jr. [11] presented a family of interface elements employing the Euler-Bernoulli and Timoshenko beam theories for the analysis of composite beams with an interlayer slip, from which the former was claimed preferable for simplicity, whereas the latter had been shown producing the most accurate structural responses, free of spurious slip strain distribution and shear locking even when high connection stiffness was considered. Due to simple modeling setting, the aforementioned linear spring-layer model had been used extensively in the study of both shear slip and weak bonding of composite laminate since it was first introduced to represent the bonding interface between laminae in Cheng et al. [12]. The trend can be observed in the following references. Dealing specifically with the geometrical effects, the sensitivity of plates, with different length-to-thickness ratios, to slightly weak shear slip was reported in [13–18] where the central deflections of stockier plates, that is, with smaller length-to-thickness ratio, are more critical than those of slender. A similar outcome was noticed in a four-layer antisymmetric cross-ply laminate with shear slip as compared to three-layer symmetric cross-ply laminate [17, 18]. By means of meshless approach and adopting the state-vector equation and the spring-layer model, Li et al. [19] examined the free vibration and eigenvalue sensitivity problems of composite laminates with interfacial imperfection where the common dependency of numerical error on the number of layers was eliminated in their model. Also, the spring-layer model has found useful applications in other imperfect layered structures such as beam [20], cylindrical panel [21, 22], stiffened plate [23], and multiferroic plate [24] where the influence of extent of imperfection as well as geometrical effects [20–22] and edge supports [23] on the bending characteristic had been discussed. Moreover, the spring-layer model had been used successfully in the study of defected smart structures (i.e., laminate, sandwich laminate, panel, cylindrical panel, and cylindrical shell with piezoelectric field) where the effects of geometric [25–30], stacking sequences [26], edges support [28], and loading environment [25, 26] had been investigated in details as well. In these studies, thicker plates were found more sensitive to the imperfection although there are few cases where thinner cross-ply laminates are more critical for various extents of imperfection [30]. In terms of boundary condition effects, the order of sensitivity with imperfection [28] can be arranged as clamped-simply support (CS) > clamped-clamped (CC) > clamped-free end (CF) while simply support-simply support (SS) > CS for hybrid/sandwich laminates and CS > SS for laminates. Under pressure loading, the deflections of both laminate and sandwich laminate increase corresponding with the increment of extent of imperfection. The contrast is observed for sandwich laminate under thermal loading. Besides, it is to be noted that the effect of imperfection is much more critical for structures under pressure loading compared to both potential and thermal loading [25, 26]. Furthermore, the sensitivity with imperfection is more obvious in angle-ply laminates with lower ply angle under pressure and potential loading as well as in intermediate ply angle for those under thermal loading [26].

 In addition to shear slip, the weak bonding modeling which includes a normal opening had been investigated by Shu and Soldatos [9], Soldatos and Shu [31], Williams [32], and Williams and Addessio [33] considering two-layer cross-ply laminates where their effects on the through-thickness midpoint deflection were studied. In this coupled condition, the thickness of laminate in relation to its surface dimensions plays a dominant role. Taking into account this particular parameter, the sensitivity of the plate, with different length-to-thickness ratios, to a complete debonding was explored in terms of midpoint deflection in Williams [32] and Williams and Addessio [33]. Moreover, the influence of different extents of bonding, ranging from a perfect bonding to complete debonding, on the midpoint bending response of laminates with *S* = 4, 10, and 100 had been highlighted in Soldatos and Shu [31]. Such an effect had also been examined in two-layer laminates with a symmetric layup [9, 31, 34–36] and an antisymmetric layup [37]. With regard to the symmetric laminate, Liu et al. [35, 36] and Soldatos and Shu [31] had reported the midpoint bending response of plate under various combinations of axial and normal imperfection, whereas a uniform degradation had been assumed in both directions in Soldatos and Shu [31]. In much similar veins, Fu et al. [38] compared in addition to intraply damage the sensitivity of the layers number of plate (*N* = 2, 4, and 6) to weak bonding, assessed in the merit of midpoint deflection, where a greater severity was found in thicker plates. From the standpoint of fiber orientation, Kam et al. [39] studied interfacial degeneration effects on the bending response of two-layer laminates, in which a generalization polar plot that incorporates numerous affecting parameters was constructed. In advanced applications, weak interface with a coupling of three fields description was proposed by Kapuria and Nair [25] and Shu [40] for predicting the behavior of piezoelectric laminates under the influence of a combination of thermal, mechanical, and electrical loads. Also, shell element has shown satisfactory performance in studying delamination in shell structures [41–43] under various boundary conditions where more generalized stacking sequences have been considered in Achryya et al. [41].

Although extensive efforts had been devoted to the study of degenerated bonding in composite structures, it should be noted that most of the aforementioned studies applied their models under relatively simple consideration, that is, 2D simply supported composite plate in presence of cylindrical bending, due largely to their use of analytical model which cannot address problem in a discreet manner. Despite simplicity in modeling, these studies were restricted to solutions in terms of 1D through-thickness responses of plate only. Also, the imperfection was usually modeled such that the bonding is degenerated uniformly throughout the whole surface area although a localized imperfection in terms of length and thickness directions, for example, mid-span/edge and top/bottom layer, respectively, had been considered in [7, 8, 20–22, 24–26, 28–30, 35, 44–47]. However, the imperfection though localized was often modeled as a debonded line extending either the width or length direction rather than that of an area [41–43], implying that the effects of imperfection are so far explored on a length-wise basis, which is not practically representative. Hence, a formulation which allows interfacial degeneration to occur partially in a localized form in terms of the plate's surface area is needed. To our knowledge, there is as yet little literature evidence on the effects of these considerations when taken simultaneously on the bending performance of laminated structures. A formulation is therefore first offered, and its associated behaviors are then discussed to fill this research gap.

In this paper, we shall permit investigation on the bending performance of two-layer cross-ply laminated plates, with an interface element in between, to handle the above mentioned considerations. The paper is arranged as follows. In the next section, the finite element formulation of a composite plate element that incorporates a virtually zero-thickness interface element will be given. The influence of localized interfacial imperfection in terms of distance, size, and extent, in the presence of two boundary conditions, simply supported and clamped, will be discussed in Section 3. Consequently, the paper ends with a summary of our main findings.

## 2. Finite Element Formulation

**Figure 1:**
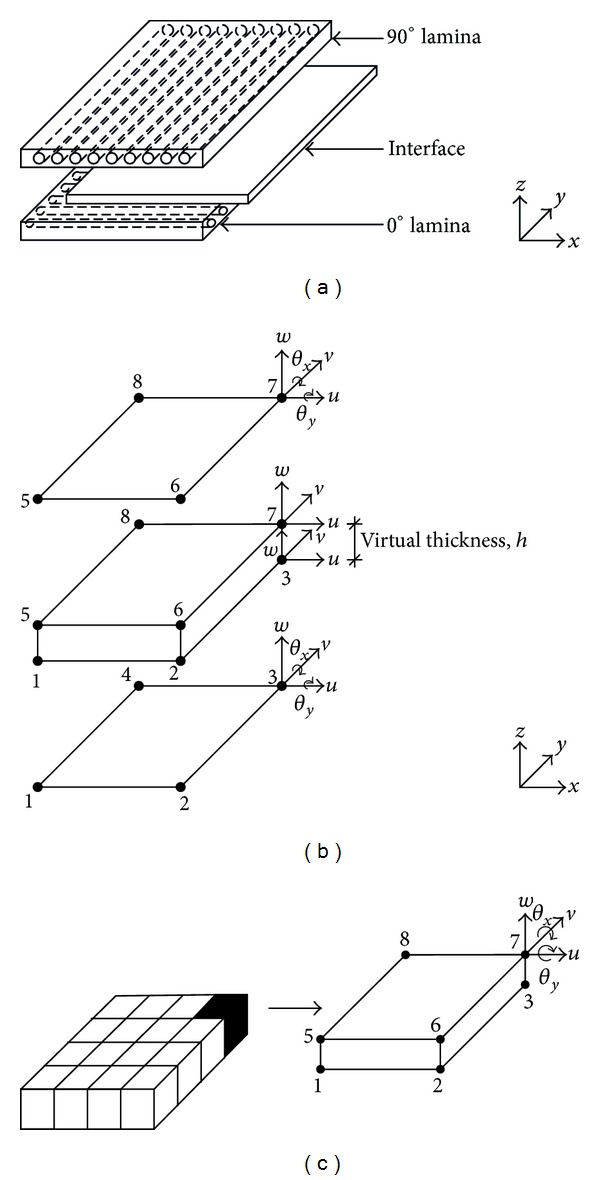
(a) Configuration of composite laminate. (b) DOF of lamina subelements and an interface element that lies in between. (c) DOF of laminate plate element, a combination of two lamina subelements, and an interface element.

Figure 1(a) shows the configuration of the [90/0] cross-ply composite laminate considered in this paper. Generally, the laminate consists of two layers of lamina with an interface layer that lies in between. In this paper, perturbation will be prescribed in the interface layer to simulate different bonding conditions, ranging from a perfect bonding to total delamination, in a localized manner. To study the influence of these conditions in composite laminate, we adopt the finite element method (FEM) in our current model. To serve our purpose, a composite plate element incorporating an interface element is therefore developed and described in the following. As a result of the introduction of an interface layer to the laminate, the computation of the stiffness matrix in current formulation differs from that of conventional. The elemental stiffness matrix of laminate is conventionally computed through the correlation of the constitutive matrix, the ABD matrix, of the laminate and the element strain-displacement matrix in the following fashion:

(1) KLAM=∬R[BiTALAMBi+BiTBLAMBo     +BoTBLAMBi+BoTDLAMBo]∂A,

where *K*_LAM[20×20]_ is the stiffness matrix of laminate, *B*_*i*[8×3]_ and *B*_*o*[12×3]_ are the in-plane and the out-of-plane element strain-displacement matrices, respectively, and *A*_LAM[3×3]_, *B*_LAM[3×3]_, and *D*_LAM[3×3]_ are the extensional, coupling, and bending stiffnesses of laminate, respectively.

It is essential to state here that the contribution of each lamina to the global behavior is readily defined in (1). As interesting as this may seem, such formulation is incapable of addressing imperfect adhesion that occurs between laminae since an assumption of perfect bonding is made in its initial formulation premise. Therefore, the remedy to this matter obligingly requires a separate formulation for the computation of stiffness matrices of lamina and interface layer. They are, respectively, elaborated as follows.

### 2.1. Stiffness Matrix of Lamina

The stiffness matrix of lamina in the current formulation is computed using a formula similar to (1). However, the stiffness matrix is computed by relating the ABD matrix of the lamina shown in (2) to the element strain-displacement matrix, replacing that of laminate as stated in (1):

(2) $$\begin{array}{c} A_{\text{lam}}=\bar{Q_{\text{lam}}^{k}}\left(z_{k}-z_{k-1}\right),\\ B_{\text{lam}}=\frac{1}{2}\bar{Q_{\text{lam}}^{k}}\left(z_{k}^{2}-z_{k-1}^{2}\right),\\ D_{\text{lam}}=\frac{1}{3}\bar{Q_{\text{lam}}^{k}}\left(z_{k}^{3}-z_{k-1}^{3}\right), \end{array}$$

where *A*_lam_, *B*_lam_, and *D*_lam_ (all [3 × 3]) are the corresponding stiffness terms, ${\bar{Q_{\text{lam}}^{k}}}_{\left[3\;\times \;3\right]}$ represents the reduced stiffness of lamina, and *z*_*k*_ is the distance of the *k*th lamina surface from the midplane of the laminate [48]. Note that the obvious difference in the current formulation is that the material stiffness terms are not assembled employing the perfect bonding assumption prior to the finite element formulation. For convenience, we replace *K*_LAM_, *A*_LAM_, *B*_LAM_, and *D*_LAM_ with *K*_lam_, *A*_lam_, *B*_lam_, and *D*_lam_, respectively, the latter terms of which are defined for lamina.

In terms of the FEM description, each lamina is modeled by a four-node lamina subelement. The corresponding arrangements of nodes and degrees of freedom (DOF) are shown in Figure 1(b). Generally, the numberings of nodes in the lamina subelement are arranged in anticlockwise manner, and each node contains 5 DOF which includes displacements in *x*-, *y*-, and *z*-directions (*u*, *v*, and *w*, resp.) as well as rotations about *y*- and *x*-directions (*θ*_*x*_ and *θ*_*y*_, resp.).

The displacements of lamina subelement are expressible as

(3) $$\begin{array}{c} u=\left[N_{i}\right]\left\{u_{i}\right\};\;\;v=\left[N_{i}\right]\left\{v_{i}\right\};\\ w=\left[N_{o}\right]\left\{w_{i}\right\}, \end{array}$$

where *N*_*i*_ and *N*_*o*_ are respectively the in-plane Lagrange shape function and out-of-plane polynomial shape function of a nonconforming rectangular element with 12 terms. In detail, {*u*_*i*_} = {*u*_1_ *u*_2_ *u*_3_ *u*_4_}^*T*^, {*v*_*i*_} = {*v*_1_ *v*_2_ *v*_3_ *v*_4_}^*T*^, and {*w*_*i*_} = {*w*_1_ *θ*_1*x*_ *θ*_1*y*_ *w*_2_ *θ*_2*x*_ *θ*_2*y*_ *w*_3_ *θ*_3*x*_ *θ*_3*y*_ *w*_4_ *θ*_4*x*_ *θ*_4*y*_}^*T*^. It follows that *B*_*i*_ = ∂*N*_*i*_ and *B*_*o*_ = ∂*N*_*o*_, where ∂ is the derivative operator. The stiffness matrices of the lamina subelements are assembled in the local stiffness matrix of the laminate element as follows:

(4) $$\begin{array}{c} K_{\text{LAM}}=\left[\begin{array}{cc} K_{\text{lower}} & K_{\text{null}}\\ K_{\text{null}} & K_{\text{upper}} \end{array}\right], \end{array}$$

where *K*_LAM[40×40]_ is the stiffness matrix of laminate element, *K*_lower[20×20]_ is the stiffness matrix of 0° lamina subelement, *K*_null[20×20]_ is the null matrix, and *K*_upper[20×20]_ is the stiffness matrix of 90° lamina subelement.

Figure 1(c) shows the DOF of the laminate plate element developed in this study. The laminate plate element consists of 8 nodes, the numberings of which are ordered in anticlockwise fashion from bottom to top, and each node possesses 5 DOF, which is similar to that of lamina subelement.

### 2.2. Stiffness Matrix of Interface

We adopt here for the interface layer a virtually zero-thickness interface element. There are eight nodes in the zero-thickness interface element, the node sequence of which is arranged in anticlockwise manner from bottom to top (refer Figure 1(b)). Each node in the zero-thickness interface element contains 3 DOF, which are represented as below:

(5) $$\begin{array}{c} \left\{d_{\text{bot}}\right\}=\left[N\right]\left\{\bar{d_{\text{bot}}}\right\};\;\;\left\{d_{\text{top}}\right\}=\left[N\right]\left\{\bar{d_{\text{top}}}\right\}, \end{array}$$

where

(6) $$\begin{array}{c} \left\{d_{\text{bot}}\right\}={\left\{\begin{array}{ccc} u_{\text{bot}} & v_{\text{bot}} & w_{\text{bot}} \end{array}\right\}}^{T},\\ \left\{d_{\text{top}}\right\}={\left\{\begin{array}{ccc} u_{\text{top}} & v_{\text{top}} & w_{\text{top}} \end{array}\right\}}^{T},\\ \left\{\bar{d_{\text{bot}}}\right\}={\left\{u_{1}\;v_{1}\;w_{1}\;u_{2}\;v_{2}\;w_{2}\;u_{3}\;v_{3}\;w_{3}\;u_{4}\;v_{4}\;w_{4}\right\}}^{T},\\ \left\{\bar{d_{\text{top}}}\right\}={\left\{u_{5}\;v_{5}\;w_{5}\;u_{6}\;v_{6}\;w_{6}\;u_{7}\;v_{7}\;w_{7}\;u_{8}\;v_{8}\;w_{8}\right\}}^{T}, \end{array}$$

{*d*_bot_} and {*d*_top_} represent the displacements of nodes located at the bottom and the top surfaces of the interface element, respectively, and the subscripts in $\left\{\bar{d_{\text{bot}}}\right\}$ and $\left\{\bar{d_{\text{top}}}\right\}$ are the nodal numbers of the interface element. Here, the Lagrange shape function for a 4-node quadrilateral element is employed for [*N*]. It should be noted that the shape function of the zero-thickness interface element in this study is a 2-dimensional Lagrange shape function rather than that of a 3-dimensional although the interface element resembles the geometrical configuration of a solid element. The same concept has been successfully applied by Coutinho et al. [49] for a 6-node triangular zero-thickness interface element, where displacements were well represented. 

Note that the interface considered in this study is an orthotropic material with null normal stresses in *x*- and *y*-directions (*σ*_*x*_ = 0 and *σ*_*y*_ = 0) and null in-plane shear stress in *x*-*y* plane (*τ*_*xy*_ = 0). Therefore, the stress-strain relationship of the zero-thickness element can be expressed as the following:

(7) $$\begin{array}{c} \left\{\sigma \right\}=\left[D_{\mathrm{int}}\right]\left\{\varepsilon \right\}, \end{array}$$

where

(8) $$\begin{array}{c} \left\{\sigma \right\}=\left\{\begin{array}{ccc} \tau_{xz} & \tau_{yz} & \sigma_{z} \end{array}\right\},\\ \left\{\varepsilon \right\}=\left\{\begin{array}{ccc} \gamma_{xz} & \gamma_{yz} & \varepsilon_{z} \end{array}\right\}, \end{array}$$

and the constitutive matrix, [*D*_int⁡_], is given by

(9) $$\begin{array}{c} D_{\mathrm{int}}=\left[\begin{array}{ccc} G_{xz}\left(1-R\right) & 0 & 0\\ 0 & G_{yz}\left(1-R\right) & 0\\ 0 & 0 & E_{z}\left(1-R\right) \end{array}\right], \end{array}$$

where *G*_*xz*_ and *G*_*yz*_ are the in-plane shear moduli, *E*_*z*_ is the Young's modulus in the *z*-direction, and *R* is the degeneration ratio that represents the extents of degeneration of interface; that is, 0 ≤ *R* ≤ 1.

Furthermore, the strains in the zero-thickness element are computed from

(10) $$\begin{array}{c} \left\{\varepsilon \right\}=\frac{1}{h}{\left\{\begin{array}{ccc} \Delta u & \Delta v & \Delta w \end{array}\right\}}^{T}, \end{array}$$

where Δ*u*, Δ*v*, and Δ*w* are the relative displacements and *h* is the virtual element thickness. Also, note that Δ*u* = *u*_top_ − *u*_bot_, Δ*v* = *v*_top_ − *v*_bot_, and Δ*w* = *w*_top_ − *w*_bot_.

In the present study, we only focus on the localized imperfection with a uniform degeneration in *G*_*xz*_, *G*_*yz*_, and *E*_*z*_ although different degradation can be imposed on one or more of these properties by using different values of *R* in [*D*_int⁡_]. From (5) and (10), the strains in the zero-thickness element are related to its nodal displacements via

(11) $$\begin{array}{c} \left\{\varepsilon \right\}=\left[B_{\mathrm{int}}\right]\left\{\overset{\wedge }{d}\right\}, \end{array}$$

where

(12) $$\begin{array}{c} \left[B_{\mathrm{int}}\right]=\frac{1}{h}\left[\begin{array}{cc} -N & N \end{array}\right],\\ \left\{\overset{\wedge }{d}\right\}={\left\{\begin{array}{cc} \bar{d_{\text{bot}}} & \bar{d_{\text{top}}} \end{array}\right\}}^{T}, \end{array}$$

and [*B*_int⁡_]_3×24_ and ${\left\{\overset{\wedge }{d}\right\}}_{24\times 1}$ are the element strain-displacement matrix and nodal displacements of the zero-thickness element, respectively. Consequently, the element stiffness matrix of the zero-thickness interface element can be computed using

(13) $$\begin{array}{c} K_{\mathrm{int}}=\iint_{R_{n}}B_{\mathrm{int}}^{T}D_{\mathrm{int}}B_{\mathrm{int}}\left|J\right|d\xi \;d\eta , \end{array}$$

where

(14) $$\begin{array}{c} J=\left[\begin{array}{cc} \frac{\partial x}{\partial \xi } & \frac{\partial y}{\partial \xi }\\ \frac{\partial x}{\partial \eta } & \frac{\partial y}{\partial \eta } \end{array}\right] \end{array}$$

is the Jacobian matrix.

The stiffness matrix of the zero-thickness interface element is assembled accordingly into the local stiffness matrix of the laminate element (Figures 1(b) and 1(c)). The DOF (*u*, *v*, and *w*) of the nodes located at the top surface of the interface element are merged with the DOF (*u*, *v*, and *w*) of the nodes of top lamina. The same is performed for the DOF of the bottom surface of the interface element and those of bottom lamina.

The complete local stiffness matrix of the laminate element will be then arranged accordingly to form the global stiffness matrix of the laminate. By discretely manipulating the properties of the zero-thickness interface element, any intensity of interfacial imperfection can be prescribed at any location of the laminate. We shall next consider the effects of various perturbations of interfacial properties on the transverse deformation of a [90/0] laminated plate using such concept.

## 3. Performance of Degenerated Laminated Composite

### 3.1. Verification

A two-layer cross-ply composite laminate with a perfect bonding and the same laminae thickness, as shown in Figure 1(a), is modeled for verification of the present model. The fiber and the matrix of the lamina are E-Glass and Epoxy (3501-6), respectively, the composite material properties of which are shown in Table 1. In addition, the material properties of the interface layer are set similar to those of the matrix since it is customarily used in practice as the bonding component for laminates. It should be noted that the thickness of the interface layer is prescribed as one-tenth of the lamina thickness in accordance with the study conducted by Sheikh and Chakrabarti [50]. 

**Figure 2:**
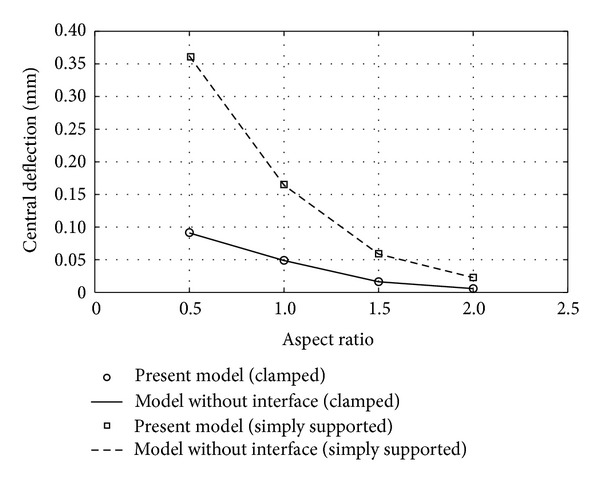
Central deflection of perfectly bonded laminate of numerous aspect ratios with simply supported and clamped boundary conditions.

The laminate is subjected to a 0.1 *μ*N/mm^2^ transversely distributed surface load and analyzed in terms of its bending behavior separately under two boundary conditions, simply supported (*u*, *v*, and *w* = 0) and clamped (*u*, *v*, *w*, *θ*_*x*_, and *θ*_*y*_ = 0), at all edges. Also, it is discretized into 16 × 16 square elements under numerous aspect ratios. The results in terms of central displacement are shown in Figure 2.

It is shown generally that the central deflections of simply supported two-layer cross-ply laminates are greater than those of clamped since the latters are constrained with a greater extent at the edges. Under the same intensity of loading and support condition, the laminate with a higher aspect ratio experiences less central deflection since it is stiffer due to close proximity of increased portion of laminate to constrained edges. It is obvious and verified that the central deflections computed from the present model when described as fully bonded match perfectly those of laminate element without interface element.

### 3.2. Localized Interfacial Imperfection on Diagonal Axis

**Figure 3:**
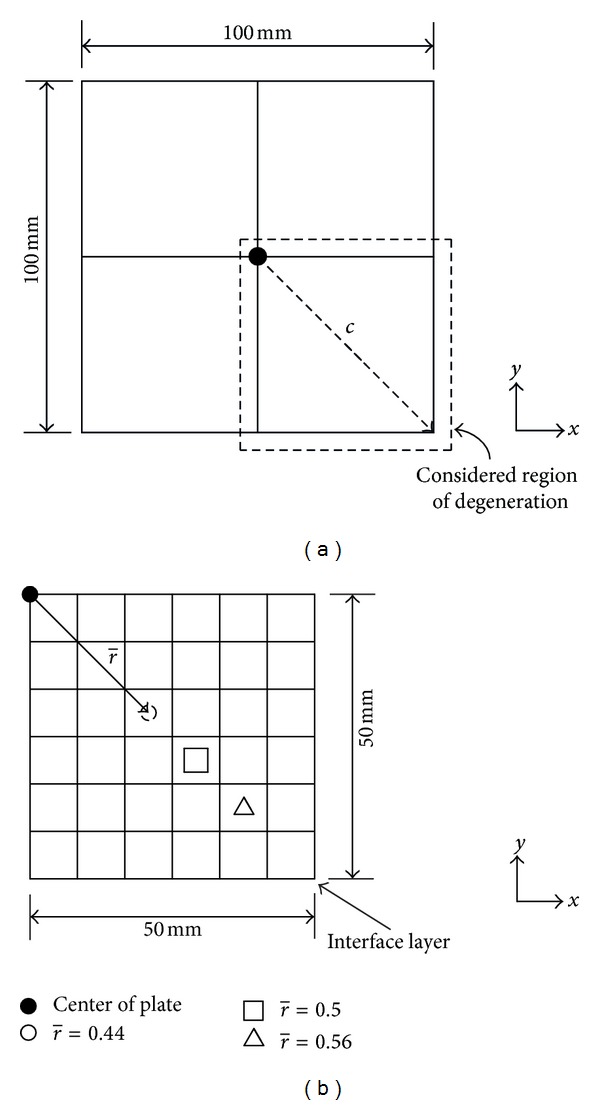
(a) Quarter of plate, the interface of which is considered for degeneration, in the analysis. (b) Localized degeneration along the diagonal of the considered region.

Departing from good agreements with the results given by the conventional FE for perfectly bonded laminates, we shall proceed, employing the current technique, to look at the bending performance of the laminate when interface is degenerated, in a variety of conditions. A composite laminate similar to that utilized in the verification (Section 3.1) will be next used to serve our purpose. All the loading and boundary conditions remain the same, except that the planar dimensions of the laminate are now fixed to 100 mm × 100 mm. Furthermore, the perturbation in the interfacial condition is focused on a quarter of the laminate, in current study, the lowest right quarter (Figure 3(a)), due to boundary condition symmetry. Besides, the localized defects simulated with different sizes and extents of imperfection are placed along the diagonal of the considered quarter plate (Figure 3(b)). The center of the localized defect, which depends on its size, may lie at the center or the edge of one of the discretized elements. Insofar as the results are concerned, we shall investigate the relative loss in structural response, when measured against its perfect state, to gain comparatively the effect of interfacial degeneration.

We define the distance of localized interfacial degeneration (*r*) as that determined from the center of plate to center of degeneration as shown in Figure 3(b). In addition, the extent of degeneration in laminate is modeled through the variation in the degeneration ratio (*R*) in the computation of the stiffness matrix of interface (9). The degeneration ratio is set to zero (*R* = 0) when the bonding is perfect and one (*R* = 1) when the laminate is debonded. In current study, we shall permit only imperfection that degrades uniformly with the same extent in *G*_*xz*_, *G*_*yz*_, and *E*_*z*_. In addition, we define the area ratio (*A*_*r*_), the distance ratio (*r*) as well as the percentage of difference using the following:

(15) $$\begin{array}{c} \text{area ratio},A_{r}=\frac{\text{surface area of localized degeneration}}{\text{surface area of laminate}}, \end{array}$$

(16) $$\begin{array}{c} \text{distance ratio},\overset{-}{r}=\frac{r}{c}, \end{array}$$

(17) $$\begin{array}{c} \text{percentage of difference}=\frac{w_{R\neq 0}-w_{R=0}}{w_{R=0}}\times 100. \end{array}$$

*c* in (16) is the length of the diagonal of the lowest right quarter, and *w*_*R*≠0_ and *w*_*R*=0_ in (17) are the central deflections of laminate with localized imperfect bonding and that of perfect bonding, respectively.

#### 3.2.1. Influences of Distance and Extent of Degeneration

**Figure 4:**
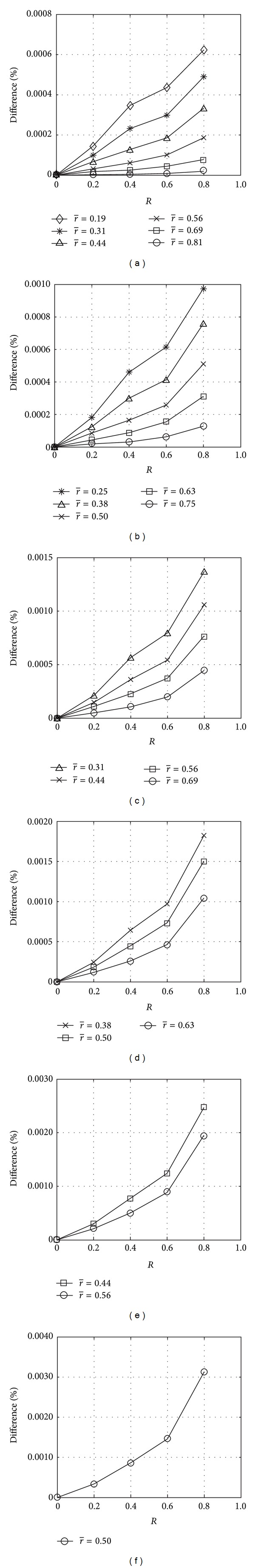
Influence of degeneration ratio (*R*) for laminates with clamped edges for (a) *A*_*r*_ = 0.0352, (b) *A*_*r*_ = 0.0625, (c) *A*_*r*_ = 0.0977, (d) *A*_*r*_ = 0.1406, (e) *A*_*r*_ = 0.1914, and (f) *A*_*r*_ = 0.2500 ($\bar{r}=0.5$).

**Figure 5:**
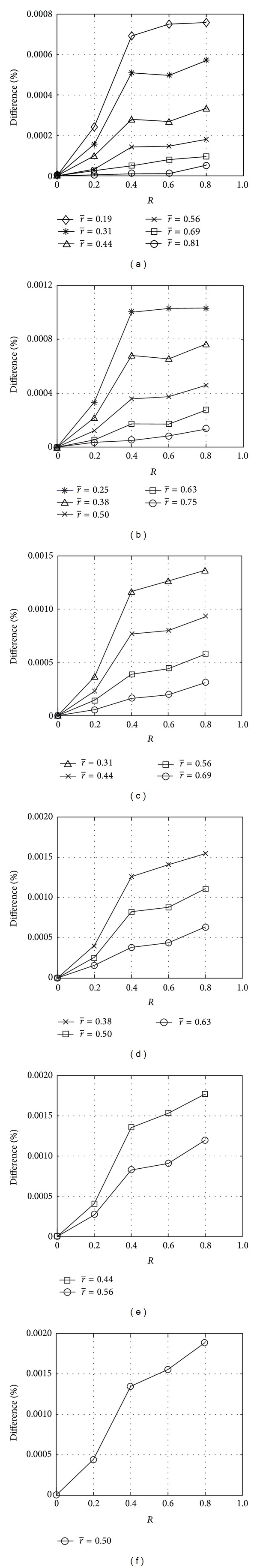
Influence of degeneration ratio (*R*) for laminates with simply supported edges for (a) *A*_*r*_ = 0.0352, (b) *A*_*r*_ = 0.0625, (c) *A*_*r*_ = 0.0977, (d) *A*_*r*_ = 0.1406, (e) *A*_*r*_ = 0.1914, and (f) *A*_*r*_ = 0.2500 ($\bar{r}=0.5$).

**Figure 6:**
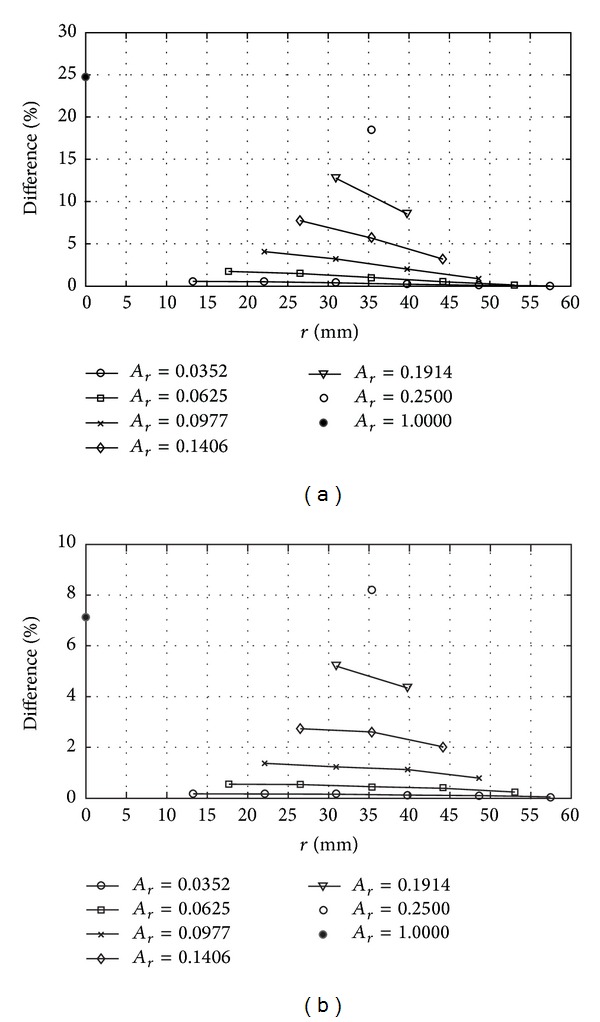
Influence of distance of total debonding (*R* = 1) on the bending behavior of laminates with (a) clamped and (b) simply supported edges for various area ratios.

The effects of variations in degeneration distance (*r*) and extent (*R*) in laminates with clamped and simply supported edges are shown in Figures 4, 5, and 6. Since greater effect is noticed in the case of *R* = 1 (total debonding) compared to *R* ≤ 0.8 (partial degeneration), the former is singled out and displayed in terms of independent plots in Figure 6. Generally, a greater percentage of difference indicates that the laminate experiences a higher central deflection with respect to that of perfectly bonded case. In other words, the localized interfacial degeneration imparts greater severity on the bending behavior of the composite laminate. Under the same *A*_*r*_ and *R*, Figures 4 and 5 show that the percentage of difference increases when the center of degeneration is approaching the center of plate, which is characterized by a smaller value of *r*. The observation is further supported by the results shown in Figure 6 where the percentage of difference rises in a similar manner. This behavior is somewhat straightforward, attributing to the closeness to supported boundaries, since the closer the considered region is to the edges, the more intense the degree of constraint for the downward translation is. As a result, a lower deflection is expected and vice versa. It should be noted that *A*_*r*_ and *R* for each curve in Figure 6 are constant, and hence, the plots solely exhibit the influence of distance of localized degeneration.

Comparing Figures 4 and 5, we notice notably distinctive trends between the clamped and simply supported laminates. As the percentage of differences for all plots of the former continually increases, when *R* rises, the latter seems to converge to particular values with a considerably reduced rate, featured by the slope of the curve especially after *R* = 0.4, although somewhat small increment is still possible. This indicates that laminates with clamped edges are relatively more sensitive to the variation in *R*. Focusing on the variation in *R*, it is evident and applicable for all cases in Figures 4 and 5 that the percentage of difference escalates corresponding to an increase in *R* for constants *A*_*r*_ and *r*, implying that a greater relative deformation is experienced when the extent of imperfection is enhanced although most deformations are practically small, up to a maximum of around 0.0032% only. It is noteworthy to see that more practically significant result can be observed in the case of *R* = 1. The pattern mimics the cases of *R* ≤ 0.8, but with greater magnitude. It is striking to notice in simply supported laminate that a total separation of a quarter region of the plate (*A*_*r*_ = 0.25) inflicts greater relative deflection than that of a fully debonded laminate (*A*_*r*_ = 1), as displayed in Figure 6. Therefore, in such case, a full degeneration of a quarter portion of a plate can be more severe in terms of deflection due to bending than a total separation of interface, the latter of which is generally accepted as the worst case scenario. We do not attempt at this stage to offer an exhaustive explanation to this peculiar result, except by stating that such condition may be linked to different load transferring efficiencies in the concerned cases. Given this phenomenal event, there exists furthermore a few insignificant drops of percentage of difference, away from the above discussed rising trend, as exhibited in Figure 5(a) (*r* = 0.31 and *r* = 0.44) and Figure 5(b) (*r* = 0.38 and *r* = 0.63) when *R* = 0.6. These inconsistencies are considered negligible in the present study due to extremely small resulting magnitude in comparison to laminates with greater interfacial degeneration. The conditions do not exist when the size of the localized imperfection increases, in which case the effect on flexural performance of the plate is much more critical and practically relevant, as readily shown in Figure 6.

#### 3.2.2. Influence of Size of Degeneration

**Figure 7:**
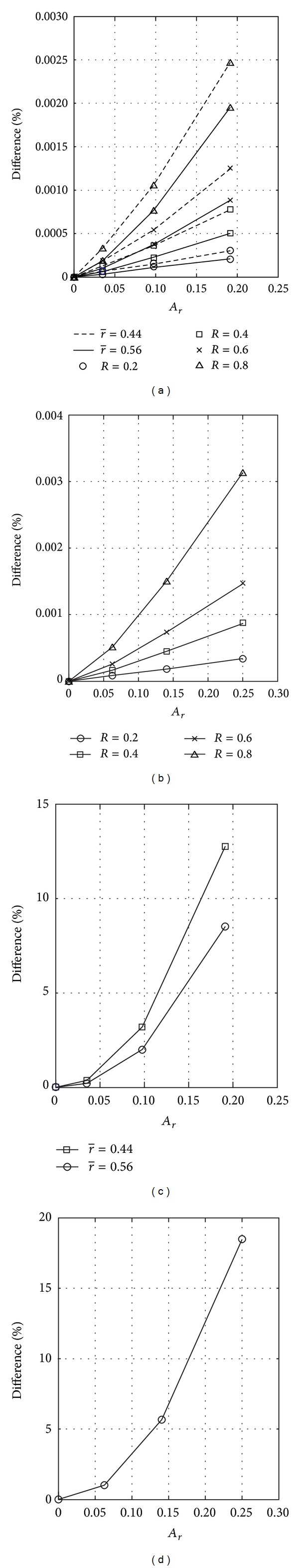
Influence of area ratio on the bending behavior at distance ratios of (a) 0.44 and 0.56, (b) 0.50, (c) 0.44 and 0.56 (*R* = 1), and (d) 0.50 (*R* = 1) from center of plate with clamped edges.

**Figure 8:**
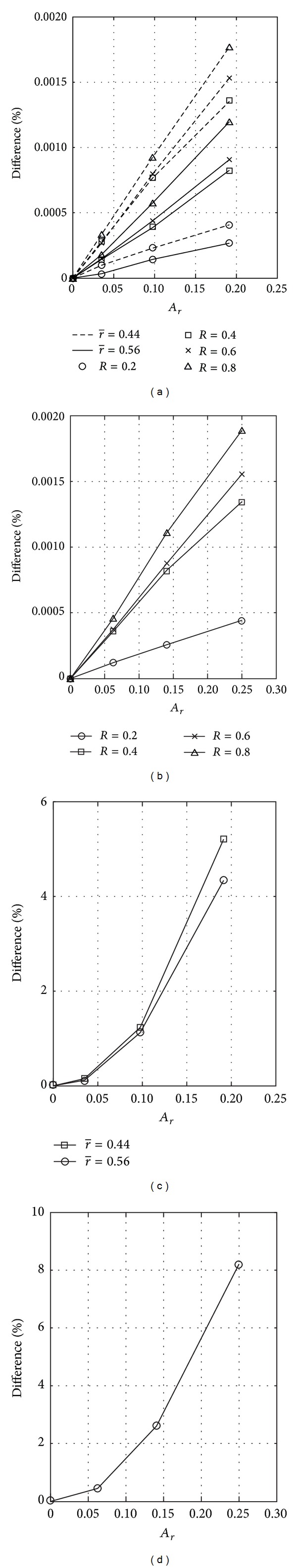
Influence of area ratio on the bending behavior at distance ratios of (a) 0.44 and 0.56, (b) 0.50, (c) 0.44 and 0.56 (*R* = 1), and (d) 0.50 (*R* = 1) from center of plate with simply supported edges.

In order to study the influence of the size of localized interfacial degeneration on the bending behavior, *r* is fixed to a constant for a variety of degeneration areas. Such condition is investigated for numerous cases of different *r*. Three degeneration distances located in the lower right quarter of the composite laminate, which include *r* = 30.9359 mm, 35.3553 mm, and 39.7748 mm, corresponding to *r* = 0.44, 0.5, and 0.56, respectively, are considered. The locations of these points are shown in Figure 3(b). The area ratios considered for the first and third *r* are 0.0352, 0.0977, and 0.1914. On the other hand, the area ratios assigned to *r* = 35.3553 mm include 0.0625, 0.1406, and 0.2500. The outcomes are shown in Figures 7 and 8, for laminates with clamped and simply supported edges, respectively.

Each curve in Figures 7 and 8 is attributed to the same *r* and *R*. Again, we have separated plots for *R* = 1 and *R* ≤ 0.8 for the same reason mentioned in Section 3.2.1. Generally, the percentage of difference grows when *A*_*r*_ increases. In other words, the influence on the central deflection of composite laminate is more significant when the size of the localized imperfection is bigger. Furthermore, the results in Figures 7(a), 7(c), 8(a), and 8(c) provide useful information in terms of the influence of the distance of localized degeneration on the bending behavior of the imperfect composite laminate. The influence of distance is best compared using the pairs of curves (dashed and solid) with the same data point symbol especially in Figures 7(a) and 8(a) where the same *R* and *A*_*r*_ are considered. It can be noticed that the plate experiences smaller relative central deflection when *r* is bigger, which represents that the localized degenerated area is further from the center of plate, as a result of close proximity to constrained edges. Meanwhile, Figures 7(a), 7(b), 8(a), and 8(b) show that the central deflection of the composite laminate is higher corresponding to an increase in *R*, replicating similar observation as presented in Section 3.2.1 although the magnitudes are considerably lesser than those with *R* = 1 as shown in Figures 7(c) and 7(d) as well as Figures 8(c) and 8(d) for clamped and simply supported edges, respectively. The responses are somewhat linear in the cases of *R* ≤ 0.8, especially for simply supported laminates. The vanishing of linearity in responses can be noticed in the case of *R* = 1. Percentages of difference are up to 13–18% and 5–8% for clamped and simply supported laminates, respectively, when *R* = 1. When *R* ≤ 0.8, they are considerably smaller in the ranges of 0.0025–0.0032% and 0.0018-0.0019%, respectively. Again, differences are markedly seen only in the case of total delamination, with an increased severity that directly corresponds to a growth in degenerated area.

On the sensitivity of boundary conditions, imperfectly bonded composite plates with all clamped edges generally experience greater relative central deflection as compared to those of simply supported. Note that the percentage of difference is merely a relative measure since it demonstrates only, in a comparative manner, the performance of plates for each respective boundary condition. In other words, clamped plates deflect more with respect to their perfectly bonded case compared to simply supported plates for the same boundary condition. Surely, simply supported plate deflects more than that of clamped when the same degeneration occurs. Therefore, it should be informed from the current finding that not only does a special attention need to be paid to the extent of interfacial imperfection, the boundary condition of the laminated composite structures is equally important since the bending behavior varies differently under these aspects.

## 4. Conclusion

A finite element formulation that integrates a virtually zero-thickness interface element is presented for studying the bending of composite laminate plates, in presence of diagonally perturbed interfacial degeneration. Employing the current model, the interfacial bonding degeneration can be inflicted discretely at arbitrary locations with various degeneration areas and intensities, under different boundary conditions, in contrast to many existing analytical models, whose degeneration occurs globally or positioned only at single site. From numerical investigation, remarkable dependencies of the bending behavior of laminate on the distance, extent, and area of the localized degeneration as well as boundary condition are exhibited. In general, a reduced bending performance correlates with increases in the degeneration extent and size but decreases in the degeneration distance from the laminate center. Also, practically significant reduction in performance is observed only in laminates with total delamination (*R* = 1) of numerous localized areas, with degradations in bending response that range from 5% to 18%. In simply supported condition, laminate with a quarter area total interfacial debonding displays strikingly the most significant degradation in performance, even greater than that with a total laminate delamination. Relatively, interfacially defected fully clamped laminates experience more severe deflection in comparison to those simply supported, when contrast against their respective fully bonded cases. Although it is outside the scope of the paper, it is interesting to note here that the current approach and methodology can be potentially implemented in the examination of other mechanical behaviors of any layered structure, in presence of various states of localized interfacial degeneration.

## Figures and Tables

**Table 1:** Material properties of lamina.

Property	Value
Longitudinal extensional stiffness, *E*_1_ [N/mm^2^]	45520
Transverse extensional stiffness, *E*_2_ [N/mm^2^]	17468
Shear modulus, *G*_12_ [N/mm^2^]	5345
Poisson's ratio, *ν*_12_	0.278
